# PABPN1 functions as a downstream gene of CREB to inhibit the proliferation of preadipocytes

**DOI:** 10.5713/ab.24.0072

**Published:** 2024-08-26

**Authors:** Xiao-Han Zhang, Jia-Xin Li, Xiao-Xu Wu, Qian Zhang, Ming Tian, Si-Qi Yang, Di Liu, Xiu-Qin Yang

**Affiliations:** 1 College of Animal Science and Technology, Northeast Agricultural University, Harbin 150030, China; 2 Institute of Animal Husbandry, Heilongjiang Academy of Agricultural Sciences, Harbin, 150086, China

**Keywords:** CREB, PABPN1, Preadipocyte, Proliferation, Regulatory Axis

## Abstract

**Objective:**

This study was conducted to reveal the role of nuclear poly(A) binding protein 1 (PABPN1) in the proliferation of preadipocytes, and to reveal the relationship between PABPN1 and cAMP response element (CRE)-binding protein (CREB) in the regulation of preadipocyte proliferation.

**Methods:**

Vectors overexpressing and siRNAs against PABPN1/CREB were transiently transfected into both porcine preadipocytes and mouse 3T3-L1 cells. Preadipocyte proliferation was measured with cell counting kit-8, 5-ethynyl-2′-deoxyuridine, real-time quantitative polymerase chain reaction, Western blotting, and flow cytometry analyses. Additionally, the transcriptional regulation of CREB on PABPN1 were analyzed with dual-luciferase reporter gene and electrophoretic mobility shift assay.

**Results:**

Overexpression of PABPN1 inhibits, and knockdown of PABPN1 promotes, the proliferation of both porcine preadipocytes and 3T3-L1 cell lines. PABPN1 overexpression increased, while knockdown decreased, the cell population in the G0/G1 phase. These indicates that PABPN1 repressed preadipocyte proliferation by inhibiting cell cycle progress. Additionally, it was revealed that CREB regulated the expression of PABPN1 through binding to the promoter and that CREB inhibited preadipocyte proliferation by repressed cell cycle progress. Furthermore, we showed that PABPN1 functions as a downstream gene of *CREB* to regulate the proliferation of preadipocytes.

**Conclusion:**

PABPN1 inhibits preadipocyte proliferation by suppressing the cell cycle. We also found that CREB could promote PABPN1 expression by binding to a motif in the promoter. Further analysis confirmed that PABPN1 functions as a downstream gene of *CREB* to regulate the proliferation of preadipocytes. These results suggest that the CREB/PABPN1 axis plays a role in the regulation of preadipocyte proliferation, which will contribute to further revealing the mechanism of fat accumulation.

## INTRODUCTION

Nuclear poly(A) binding protein 1 (PABPN1) is an RNA binding protein and implicated in multiple steps of RNA metabolism through binding to poly(A) tail of mRNA. It was associated with various post-transcriptional regulatory processes including splicing [1,2], alternative polyadenylation (APA) [3,4], and mRNA decay [5]. PABPN1 was also involved in the transportation of mature mRNA into the cytoplasm [6], and in the pioneer round of translation [7], thus playing a role in translational regulatory processes. In addition to function in the protein-coding RNA, PABPN1 has been found to promote RNA exosome-mediated turnover of long non-coding RNA (lncRNA) via a polyadenylation-dependent mechanism [8].

PABPN1 has been directly involved in some physiological and pathological processes. A short expansion of alanine tract (from 10 alanines to 12–17 alanines) in the N-terminal domain of PABPN1 polypeptide results in the oculopharyngeal muscular dystrophy, the muscle specific disease [9]. Some studies revealed that PABPN1 regulates cell proliferation. It has a promoting effect on the proliferation of primary mouse myoblasts obtained from pharyngeal, extraocular and limb muscles in which PABPN1 knockdown significantly inhibits the proliferation [10]. In tumor cells, PABPN1 exerts dual action on proliferation: it affects the progression of hepatoblastoma (HB) and clear cell renal cell carcinoma (ccRCC) by promoting cell proliferation [11,12], while inhibits the proliferation of human alveolar adenocarcinoma cell A549-tTA and bladder cancer (BC) cells [13,14]. These results indicate that the role of PABPN1 in the proliferation is diverse and cell type-specific.

The cAMP response element (CRE)-binding protein (CREB), a transcription factor recognizing CRE motif, is frequently involved in cell proliferation. And its role on cell proliferation depends highly on cell types and physiological situation. CREB contributes to inhibiting the proliferation of ovarian granulosa cells in pigs [15]. CREB also showed a negative effect on cell proliferation in Hodgkin Lymphoma as its downregulation enhances cell proliferation via mediating G1/S phase transition [16]. While in human pulmonary endothelial cells, binding of CREB at the CRE motif is crucial for hypoxia-induced expression of Gremlin1, which, in turn, promotes cell proliferation [17]. CREB has also been implicated in the proliferation of cells including vascular smooth muscle cells [18,19], hepatocytes [20], bone marrow mesenchymal stem cells [21], etc.

In the previous study, we have showed that CREB transcriptionally regulates the expression of *PABPN1* gene in pigs [22] and that PABPN1 was differentially expressed in porcine muscles with differential fat contents [23], indicating a role of PABPN1 in fat accumulation. But by far, no direct relationship between CREB/PABPN1 and preadipocyte proliferation was found. In this study, we aimed to investigate the role of PABPN1 on preadipocyte proliferation. The results showed that PABPN1 functions as a downstream gene of *CREB* to inhibit the proliferation of preadipocytes. The results will contribute to further revealing the mechanisms underlying the effects of CREB/PABPN1 axis on the preadipocyte proliferation, which will help to control human fat content and benefit human health now that obesity is becoming more common.

## MATERIALS AND METHODS

### Institutional review board statement

The animal study protocol was approved by the Animal Care Committee of Northeast Agricultural University (protocol code NEAUEC20220201, March 10, 2022).

### Animals, nucleic acid isolation, and cDNA synthesis

Min pigs at the age of 30 days were provided by the Institute of Animal Husbandry, Heilongjiang Academy of Agricultural Sciences, Harbin, China. *Longissimus dorsi* muscle was collected immediately after the pigs were slaughtered, and snap frozen in liquid. At the same time, back fat was collected for isolation of preadipocytes. Total RNA was isolated by using TRIzol reagent (Invitrogen, Carlsbad, CA, USA). Reverse transcription was performed with the HiScript III 1st Strand cDNA Synthesis Kit (+gDNA wiper) (Vazyme, Nanjing, China). All procedures of animal treatment were strictly based on the protocol of the Animal Care Committee of Northeast Agricultural University.

### Plasmids and siRNA sequences

Plasmids overexpressing CREB and reporter genes containing porcine PABPN1 promoter, wild type or mutant one deleting CREB binding site, were generated previously [22]. The complete coding sequences (CDS) of porcine PABPN1 were amplified with Taq HS Perfect Mix (TaKaRa, Dalian, China) and cDNA template obtained from pig Longissimus dorsi muscle. The polymerase chain reaction (PCR) products were inserted into pCMV-HA vector at the enzyme sites *Hind*III and *Kpn*I to construct vector overexpressing PABPN1. siRNAs against CREB were effective in both pigs and mice, were designed previously [23]. siRNAs against PABPN1 were designed separately for pigs and mice, and synthesized by General Biol (Hefei, China). The sequences were listed in Supplementary Table S1.

### Cell culture and transfection

Porcine preadipocytes were cultured as described previously [23]. Briefly, backfat tissues were cleaned and digested with 0.1% type I collagenase (Invitrogen, USA). The obtained preadipocytes were cultured in Dulbecco’s modified eagle’s medium/Nutrient Mixture F-12 (DMEM/F12) containing 10% fetal bovine serum (FBS; Gibco, Carlsbad, CA, USA) and 1% penicillin-streptomycin (Invitrogen, USA). The cells were cultured at 37°C with 5% CO_2_ and the medium was changed every 48 h. 3T3-L1 [24] and HEK-293T [25] cells were cultured as described elsewhere. Transient transfection was performed with Lipofectamine 2000 reagent (Invitrogen, USA) according to the manufacturer’s instructions.

### Cell counting kit 8 assay

Cells were inoculated in 96-well plates at a density of 5×10^3^. At 50% confluence of preadipocytes or 70% confluence of 3T3-L1 cells, the cells were transfected with overexpression plasmids or siRNA for 24 h. The cells were then collected at each time point indicated in section Results and subjected to cell counting kit-8 (CCK-8; Beyotime, Shanghai, China) assay according to the manufacturer’s instructions. The optical density (OD) was analyzed at 450 nm using an Infinite F50 Micro-plate Reader (Tean GENios, Mannendorf, Switzerland).

### 5-Ethynyl-2′-deoxyuridine incorporation assay

5-Ethynyl-2′-deoxyuridine (EdU) staining was performed with BeyoClick EdU-488 kit (Beyotime, China) according to the manufacturer’s instructions. Briefly, 2800 cells were inoculated into a 96-well plate and cultured at 37°C for 14 h. Plasmids or siRNAs were then transfected into the cells for 24 h. Cells were transferred into medium containing 10 μM EdU and cultured for another 2 h. After fixed and permeabilized, the cells were incubated with Click Additive solution for 30 min at room temperature. The cells were then stained with Hoechst 33342 for 10 min and observed with an Olympus inverted fluorescence microscope IX71 (Olympus, Tokyo, Japan). The fluorescence at 346 nm (excitation)/460 nm (emission) were measured.

### Western blotting

Western blotting was performed as described previously [26]. Briefly, cells were seeded into six-well plates, and were transfected with an overexpression vector or siRNAs. At 48 h post-transfection, total protein was isolated using RIPA buffer (Beyotime, China) supplemented with a protease inhibitor (Invitrogen, USA) and quantified with enhanced BCA protein assay kit (Beyotime, China). A total of 25 to 30 μg total protein was separated on sodium dodecyl sulfate-polyacrylamide gel, and then transferred onto a polyvinyl difluoride membrane (Millipore, Shanghai, China). Membranes were blocked with 5% skimmed milk and incubated with anti-hemagglutnin (HA) tag (1:5,000 dilution; Abmart, Shanghai, China), anti-proliferating cell nuclear antigen (PCNA) (1:5,000 dilution; Proteintech, Wuhan, China), anti-β-actin (1:3,000 dilution; Abmart, China), and anti-β-tubulin (1:1,000 dilution; Abmart, China) primary antibodies. β-actin and β-tubulin were used as control. Membranes were probed with goat anti-mouse immunoglobulin G secondary antibody (1:20,000 dilution; LI-COR, Lincoln, NE, USA). The results were detected on UVP ChemStudioTM PLUS touch (Analytik Jena, Upland, CA, USA). Gray value was analyzed with ImageJ software (version 1.51j8) to measure relative intensities of the bands.

### Real-time quantitative polymerase chain reaction

Real-time quantitative PCR (qPCR) was conducted with ChamQ Universal SYBR qPCR Master Mix (Vazyme, China) according to the manufacturer’s instructions, each with triplicates. The data was analyzed with 2^–ΔΔCt^ method using β-actin as a reference [27]. Primers were synthesized by Beijing Genomics Institute (BGI, Beijing, China) and the sequences are listed in Supplementary Table S2.

### Flow cytometry analysis

Cells were seeded in six-well plates at a density of 1.2×10^5^ per well. When reached 50% confluence, the cells were transfected with plasmids or siRNAs. At 24 h post-transfection, cells were washed twice with phosphate-buffered saline (PBS), digested with trypsin-ethylene diamine tetraacetic acid for 1 min, and resuspended with high-glucose DMEM. Then the cells were collected, and stained with cell cycle staining Kit (MultiSciences, Hangzhou, China). The cell cycle was analyzed with BD FACSCelesta (Becton Dickinson, Franklin Lakes, NJ, USA) or Agilent NovoCyte (Palo Alto, CA, USA) Flow Cytometer.

### Dual-luciferase reporter gene analysis

Each reporter gene was transfected into 3T3-L1 cells individually or together with plasmids overexpressing CREB. pRL-TK, a Renilla luciferase reporter, was used as an internal reference to avoid differences in transfection efficiency among groups. At 48 h post-transfection, the cells were collected, and subjected to analyzing luciferase activity with a dual-luciferase reporter gene assay kit (Beyotime, China). The relative luciferase activity was measured as a ratio of firefly to Renilla luciferase value.

### Electrophoretic mobility shift assay

Electrophoretic mobility shift assay (EMSA) was performed with Chemiluminescent kit (Beyotime, China) as described previously [25]. Briefly, nuclear extracts were isolated with a kit (Solarbio, Beijing, China) from HEK-293T cells. The biotin-labeled probes for CREB-specific binding and unlabeled specific competitor, a mutant competitor were synthesized by General Biol (China). The biotin-labeled wild type and mutant competitor were incubated with nuclear extracts for 20 min. The specific competitor was first incubated with nuclear extracts for 10 min before adding biotin-labeled probes and antibody extracts for 15 min. The mixture was electrophoresed on 6.5% polyacrylamide gel for 1.5 h at 90 V and then transferred to nylon membrane (Beyotime, China). The gels were observed on Azure c300 Gel Imaging System (Azure Biosystems, Dublin, CA, USA). The probe sequences are given in Supplementary Table S3.

### Chromatin immunoprecipitation (ChIP)-qPCR

The chromatin immunoprecipitation-real-time quantitative PCR (CHIP-qPCR) was conducted with SimpleChIP Enzymatic Chromatin IP Kit (Cell Signaling Technology, Beverly, MA, USA). Briefly, 2×10^7^ cells overexpressing CREB were crosslinked in formaldehyde with a final concentration of 1% for 10 min at room temperature, followed by 10×glycine treatment to terminate the crosslinking reaction. Then the cells were collected with 2 mL ice-cold PBS+protease inhibitor cocktail (PIC) and centrifuged at 2,000×g for 5 min at 4°C to obtain the nuclear pellet. After shivered to a length of approximately 150 to 900 bp by Ultrasonic Homogenizer, DNA was incubated with anti-HA tag (Proteintech, China) overnight at 4°C with rotation. Finally, qRT-PCR was used to quantify the immunoprecipitated DNA. The primers used for ChIP-qPCR are listed in Supplementary Table S2.

### Statistical analysis

All experiments were performed in triplicate. Statistical analyses were conducted with GraphPad Prism (version9.5.1; GraphPad, San Deigo, CA, USA), and unpaired t-test was used to compare the differences between groups.

## RESULTS

### PABPN1 is involved in the proliferation of preadipocytes

**Figure 1:**
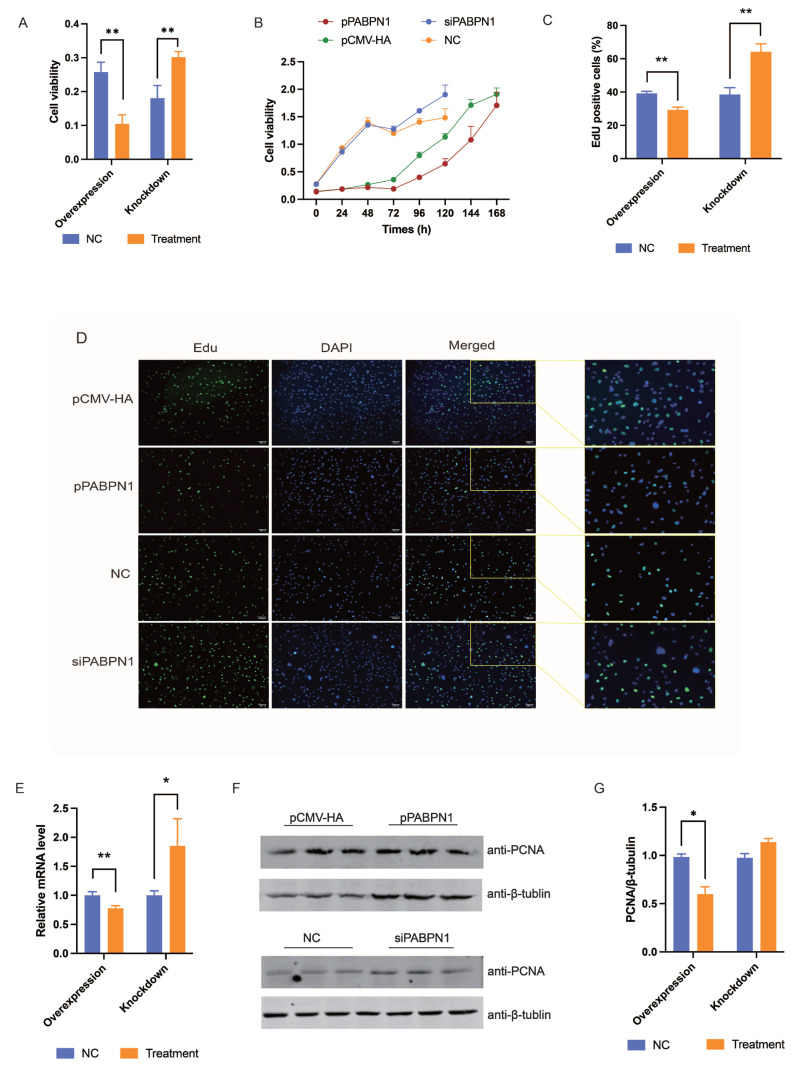
PABPN1 inhibits the proliferation of preadipocytes. (A and B) Effects of PABPN1 on the proliferation of porcine preadipocytes (A) and 3T3-L1 cells (B) as revealed by CCK-8 assay. (C and D) Effects of PABPN1 on the proliferation of 3T3-L1 cells as revealed by EdU staining after PABPN1 transfection 24 h. (E) Effects of PABPN1 on the mRNA expression of PCNA was detected 48 h after 3T3-L1 cells were transfected. (F and G) Effects of PABPN1 on the protein expression of PCNA in 3T3-L1 cells after transfected 48 h. The results were expressed as mean±standard error of the mean (n = 3). PABPN1, nuclear poly(A) binding protein 1; CCK-8, cell counting kit-8; Edu, 5-ethynyl-2′-deoxyuridine. * p<0.05; ** p<0.01.

Overexpression vector and siRNA against PABPN1 function efficiently in primary cultured porcine preadipocyte as revealed by real-time PCR and western blotting (Supplementary Figure S1A and S1B). Overexpression of PABPN1 inhibited the proliferation of porcine preadipocytes (p<0.01), and knockdown of PABPN1 promoted cell proliferation (p<0.01), by CCK-8 assays (Figure 1A), indicating a negative role of PABPN1 on porcine preadipocytes.

3T3-L1 cell lines were then used to further reveal the role of PABPN1 on preadipocyte proliferation. siRNA against mouse PABPN1 was designed successfully (Supplementary Figure S1C). Overexpression of PABPN1 represses (p<0.05), and knockdown of PABPN1 enhances (p<0.05), the proliferation of 3T3-L1 cells as revealed by CCK-8 assays (Figure 1B), and consistent results were obtained by EdU staining (p<0.01) (Figure 1C and 1D). Additionally, the expression of PCNA, which is a marker gene of cell proliferation, was decreased by overexpressing PABPN1 (p<0.01) and increased by knocking down PABPN1 (p<0.05) at both mRNA and protein levels (Figure 1E–1G).

### PABPN1 regresses preadipocyte proliferation by inhibiting cell cycle progress

**Figure 2:**
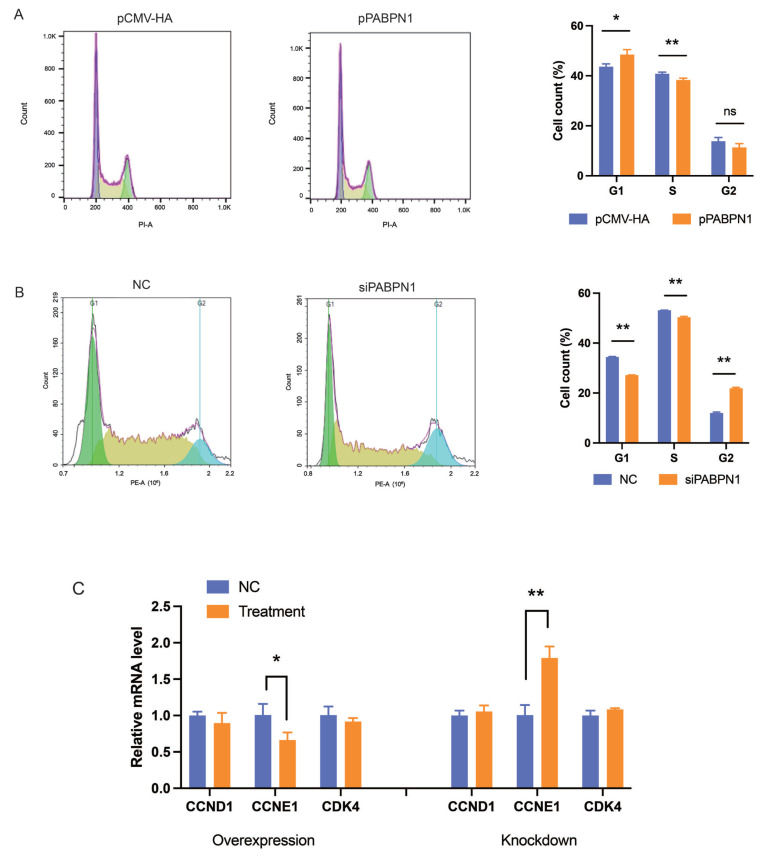
PABPN1 inhibits cell cycle progress. (A and B) Effects of PABPN1 overexpression (A) and knocking down (B) on cell cycle progress in 3T3-L1 cells after transfected 24 h. (C) Effects of PABPN1 on the ex-pression of cell cycle genes in 3T3-L1 cells were transfected overexpression and knockdown after 48 h. The results were expressed as mean±standard error of the mean (n = 3). PABPN1, nuclear poly(A) binding protein 1. ^ns^ p>0.05; * p<0.05; ** p<0.01.

To explore the mechanisms underlying the role of PABPN1 on preadipocyte proliferation, cell cycle distribution analysis was performed with flow cytometry in 3T3-L1 cells. As shown in Figure 2A and B, PABPN1 overexpression increased the cell population in the G0/G1 phase compared to that in the negative control (NC) groups (p<0.05), while PABPN1 knockdown decreased it (p<0.01). This indicated that PABPN1 inhibits cell division by arresting cells at the G0/G1 phase. Among three cell cycle genes, such as cyclin D1 (*CCND1*), cyclin E1 (*CCNE1*), and cyclin-dependent kinase 4 (*CDK4*), the expression of *CCNE1* was regulated significantly by PABPN1 (p<0.05). Overexpression of PABPN1 inhibited the mRNA level of *CCNE1* (p<0.05), while knocking down of PABPN1 increased, the mRNA level of *CCNE1* (p<0.01; Figure 2C).

### CREB regulates the transcription of PABPN1 by binding to the promoter

**Figure 3:**
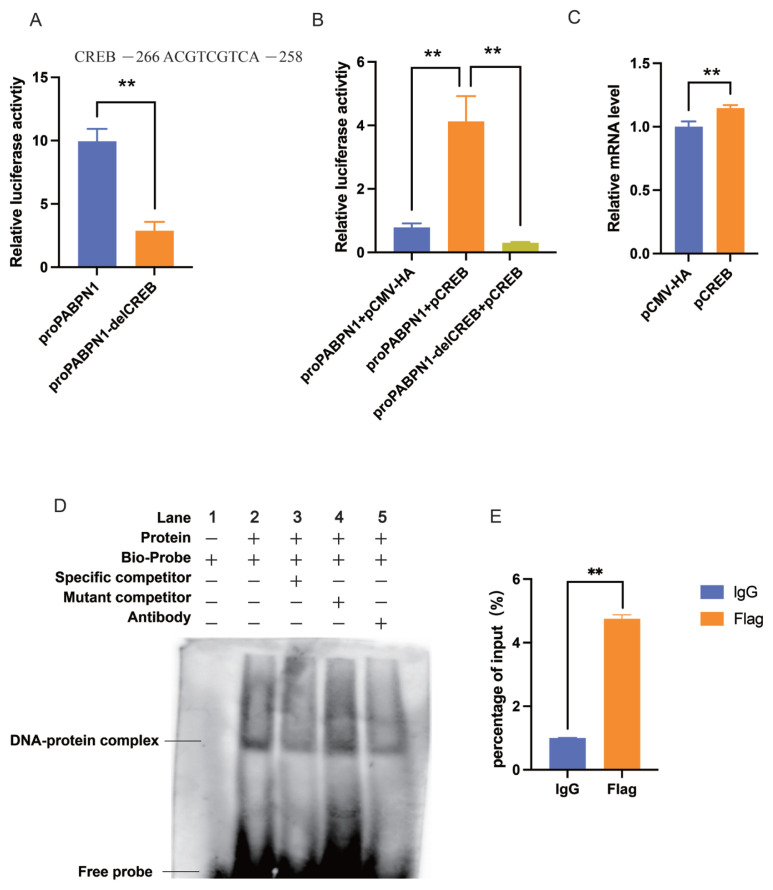
CREB promotes the expression of PABPN1 via a motif on the promoter. (A) Deletion of binding site for CREB decreases the promoter activity of PABPN1. The putative CREB motif were given above, and italic letters were deleted in the mutant-type reporter gene. (B) Ectopic CREB promotes the expression of PABPN1, and deleting the binding site reverse the promoting effect of ectopic CREB. (C) mRNA level of endogenous PABPN1 is increased by CREB overexpression. (D) CREB binds to the promoter of PABPN1 through the putative sites as revealed by EMSA assay. (E) ChIP-qPCR assay revealed the potential binding sites of CREB in the PABPN1 promoter region. The results were expressed as mean±standard error of the mean (n = 3). CREB, cAMP response element (CRE)-binding protein; PABPN1, nuclear poly(A) binding protein 1; CCK-8, cell counting kit-8; EMSA, electrophoretic mobility shift assay; ChIP-qPCR, chromatin immunoprecipitation-real-time quantitative polymerase chain reaction. ** p<0.01.

In the previous study, we showed that PABPN1 harbors one putative binding site of CREB in an adjacent promoter and that CREB can promote the expression of PABPN1 in porcine PK-15 cells preliminarily [22]. To further characterize the transcriptional regulation of CREB on PABPN1, dual-luciferase reporter gene analysis was performed in 3T3-L1 cells, and consistent results were obtained. The promoter of porcine PABPN1 can drive the expression of the firefly luciferase gene effectively and deletion of the putative site for CREB repressed the promoter activity (p<0.01) (Figure 3A). Overexpression of CREB enhanced the promoter activity (p<0.01), while the absence of the site reverses the promoted effects of CREB (p<0.01) (Figure 3B). Furthermore, ectopic CREB increased the endogenous expression of PABPN1 in 3T3-L1 cells (p<0.01) (Figure 3C). These indicate that CREB can also activate the expression of PABPN1 in mice.

EMSA analysis showed that, in the site for CREB binding, biotin-labeled probe can form DNA-protein complexes with nuclear extracts (Lane 2), and addition of mutant competitor did not affect the complex formation (Lane 4), and the complexes were weakened robustly by the specific competitor group (Lane 3). Additionally, the binding of CREB protein with antibody decreases the DNA-protein complexes in the super-shift assay (Lane 5) (Figure 3D). The results indicated CREB could bind to the sites in the promoter of *PABPN1* gene. Finally, to further prove CREB can participate in the transcriptional regulation of PABPN1, we conducted the ChIP experiment. The ChIP-qPCR also showed that CREB had significant enrichments in the PABPN1 promoter region (Figure 3E).

### CREB represses cell proliferation by inhibiting cell cycle progress

**Figure 4:**
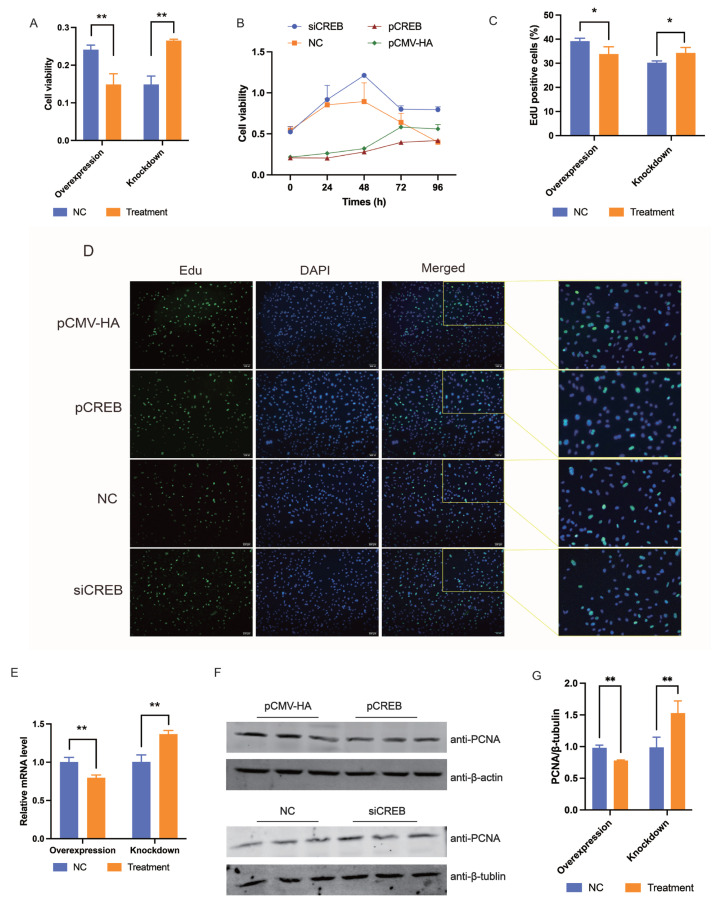
CREB inhibits the proliferation of preadipocytes. (A and B) Effects of CREB on the pro-liferation of porcine preadipocytes and 3T3-L1 cells as revealed by CCK-8 assay. (C and D) Effects of CREB on the proliferation of 3T3-L1 cells as revealed by EdU staining after transfected 24 h. (E) Effects of CREB on the mRNA expression of PCNA was detected 48 h after 3T3-L1 cells were transfected. (F and G) Effects of CREB on the protein ex-pression of PCNA in 3T3-L1 cells after transfected 48 h. The results were expressed as mean±standard error of the mean (n = 3). CREB, cAMP response element (CRE)-binding protein; PABPN1, nuclear poly(A) binding protein 1; CCK-8, cell counting kit-8; EdU, 5-ethynyl-2′-deoxyuridine; PCNA, proliferating cell nuclear antigen. * p<0.05; ** p<0.01.

Next, we evaluated the role of CREB on preadipocyte proliferation. Overexpression vector and siRNA against CREB work well in both pig and mouse cells (Supplementary Figure S2A–S2C). CREB repressed the proliferation of porcine preadipocytes as revealed by gain- and loss-of function analysis (p<0.01) (Figure 4A). In 3T3-L1 cell lines, inhibitory effects of CREB on cell proliferation were also observed via CCK-8 and EdU staining assays (Figure 4B–4D); the expression of PCNA was suppressed by CREB overexpression and promoted by CREB knockdown at both mRNA and protein level (p<0.01) (Figure 4E and 4F).

**Figure 5:**
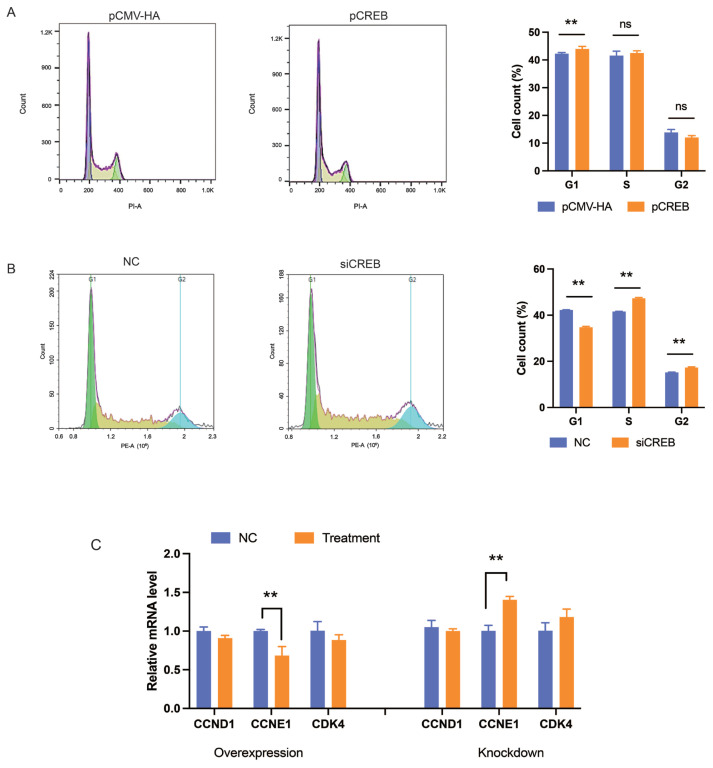
CREB inhibits cell cycle progress. Effects of CREB overexpression (A) and knocking down (B) of CREB on cell cycle progress in 3T3-L1 cells after transfected 24. (C) Effects of CREB on the expression of cell cycle gene in 3T3-L1 cells were transfected overexpression and knockdown after 48 h. The results were expressed as mean±standard error of the mean (n = 3). CREB, cAMP response element (CRE)-binding protein. ^ns^ p>0.05; ** p<0.01.

Flow-cytometry analysis indicated that CREB results in cell cycle arrest at the G0/G1 phase in 3T3-L1 cell lines (Figure 5A and 5B). Additionally, the expression of *CCNE1* gene was inhibited by CREB overexpression (p<0.05) and promoted by CREB knockdown (p<0.01), showing a negative effect of CREB on the expression of CCNE1 (Figure 5C).

### PABPN1 functions as a downstream gene of *CREB* in the regulation of preadipocyte pro-liferation

**Figure 6:**
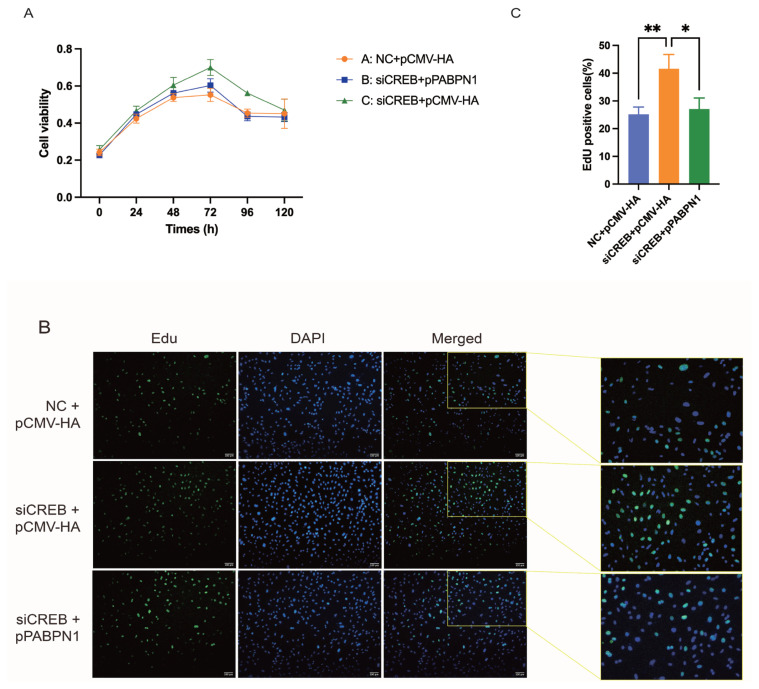
PABPN1 functions as a downstream gene of CREB to regulate cell proliferation as revealed in 3T3-L1 cells. (A) Effects of transfected overexpression of PABPN1 after CREB knockdown in 3T3-L1 cells as revealed by CCK-8 assay. (B and C) Effects of transfected overexpression of PABPN1 after CREB knockdown in 3T3-L1 cells after 24 h analysis as revealed by EdU staining analysis. The results were expressed as mean±standard error of the mean (n = 3). PABPN1, nuclear poly(A) binding protein 1; CREB, cAMP response element (CRE)-binding protein; CCK-8, cell counting kit-8; EdU, 5-ethynyl-2′-deoxyuridine. * p<0.05; ** p<0.01.

Rescue experiments were performed to reveal the relationship of CREB and PABPN1 in the regulation of cell proliferation in 3T3-L1 cell lines. CCK-8 assays showed that knock downing of CREB promotes cell proliferation compared to NC group, while cotransfection of siRNA against CREB and plasmids overexpressing PABPN1 abolished the promoting effects of CREB knockdown (Figure 6A). EdU staining showed that CREB knockdown promotes DNA replication, while the promoting effects were reversed by overexpressing PABPN1 simultaneously (Figure 6B and 6C). These results indicate that CREB regulates preadipocyte proliferation by controlling the expression of PABPN1 and that PABPN1 functions as a downstream gene of CREB.

## DISCUSSION

Cell proliferation, regulated by a complex gene expression program, is crucial for the development and survival of a multicellular organism. The proliferation of different types of cells not only occurs at different time but is regulated specifically. The factors involved in cell proliferation are changed with cell types. We make clear that both CREB and PABPN1 inhibit the proliferation of preadipocytes, and that PABPN1 functions as a downstream gene of CREB to repress preadipocyte proliferation in both primary porcine preadipocytes and 3T3-L1 cell lines. The results will contribute to revealing the mechanisms underlying preadipocyte proliferation, and to controlling the fat accumulation.

Preadipocyte proliferation is important for adipose development, and many efforts have been made to reveal the underlying mechanisms. Until now series of transcription factors [28–30], and candidate genes [31–33] were found to regulate the preadipocyte proliferation. Non-coding RNAs including miRNA and lncRNA were also involved in the process [34–36]. However, preadipocyte proliferation is complicated and highly regulated, there are still many regulators remaining to be identified. Here, *PABPN1* was selected as candidate gene for preadipocyte proliferation based on results obtained previously [22,23].

PABPN1 is highly conserved among mammals [37]. Sequence alignments showed that the identities of pig PABPN1 protein (MH795126) with that in humans (NM004643), cattle (NM174569), and mice (NM019402) are 100%, 99%, and 97%, respectively, and no alteration was found in the functional domain. This suggests a similar role of the protein among species. Thus, both primary cultured porcine preadipocytes and mouse preadipocyte cell line, 3T3-L1, were used to reveal the effect of PABPN1 on cell proliferation, and consistent results were obtained by CCK-8 assay. There are no immortalized preadipocytes to replace primary cells for further analysis in pigs. The primary cells are limited in number because they can be passaged just 3 to 4 times, and backfat tissues are small in piglets, so the cells used in the experiment cannot be guaranteed to be from the same source, resulting in heterogenicity of the cells cultured in different batches. Additionally, the fact that fat tissue is composed of multiple cells also leads to heterogenicity. All these might affect the consistency and accuracy of the results. Thereafter further experiments were performed only in 3T3-L1 cells after CCK-8 analysis. Through multiple experiments including CCK-8 assay, EdU staining, and quantitative analysis of proliferation marker gene, we showed that PABPN1 inhibits the proliferation of preadipocytes.

Cell cycle progress, regulated orderly and strictly, is the basis for cell proliferation. Cyclins and CDKs are essential for the correct progression of the process [38,39]. Once expressed, the cyclin proteins will form complexes with CDKs to activate their kinase activity, which then promotes cell cycle progression [40]. To investigate the mechanisms underlying the inhibitory effects of PABPN1 on preadipocyte proliferation, we first revealed that PABPN1 leads to G1/S phase arrest with flow cytometry. CCND and CCNE express at early and middle stage of G1 phase, respectively, and function to determine the transition of cells from G1 to S phase. Thereafter, CCNE1, CCND1, and CDK4 was selected to analyze with real-time quantitative PCR, which showed that CREB represses the expression of CCNE1. CCNE is a rate-limiting factor in G1/S phase transition. The down-regulation of CCNE1 could result in G1/S phase arrest. Thus, we demonstrated that CREB regresses preadipocyte proliferation by inhibiting cell cycle progress during which CCNE1 is an important effector.

CREB is a general transcription factor and highly conserved among species. It is estimated that more than 4,000 genes harbor CRE motif through genome-wide screening, thus might be activated by CREB [41]. Based on these huge number of target genes, CREB is associated with various physiological processes, and has been extensively involved in the proliferation of cells including ovarian granulosa cells [15], pulmonary endothelial cells [17], and tumor cells [16], etc. [18–21], showing a potential for regulating preadipocyte proliferation. We have found that CREB can promote the expression of PABPN1 in pigs previously [22]. Here, we further confirmed that CREB is a transcription factor of PABPN1 through studies in 3T3-L1 cells and EMSA. These indicate that CREB might have a role in the proliferation of preadipocyte regulated by PABPN1. The following experiments was then performed and made clear that PABPN1 inhibit the proliferation of preadipocytes as a downstream gene of *CREB*. To the best of our knowledge this is the first report on the CREB/PABPN1 axis in cell proliferation.

PABPN1 is a general player of APA, a crucial process in the post-transcriptional regulation of eukaryotic mRNAs [42]. APA is caused by the existence of multiple poly(A) signals in the same transcript and produces multiple mature mRNAs with differences in the length of 3’ untranslated region (UTR) [43]. It has been revealed that APA is critical for mRNA stability, localization, and translation [44]. Studies in ccRCC and BC cells revealed that PABPN1 regulates cell proliferation via controlling APA of target genes. Overexpression of PABPN1 prolonged the 3’ UTR of *CCND3* gene by modulating APA, decreasing the expression of CCND3 in BC cells [10]. PABPN1 also suppresses the APA of *SGPL1* and *CREG1* gene and promote ccRCC cell proliferation [28]. In HB cells PABPN1 interacts with splicing machinery and functions as an oncofetal splicing regulator [45]. Here, we showed that PABPN1 suppresses the expression of *CCNE1* to inhibit preadipocyte proliferation. However, the mechanisms underlying the regulation of PABPN1 on *CCNE1* remains to be identified.

## CONCLUSION

Here PABPN1 is first involved in the proliferation of preadipocytes. It represses preadipocyte proliferation through inhibiting cell cycle. We also show that CREB can promote the expression of PABPN1 via binding to a motif in the promoter and has a similar role to PABPN1 during preadipocyte proliferation. Further analyses confirm that PABPN1 functions as a downstream gene of CREB to modulate preadipocyte proliferation. These results indicate CREB/PABPN1 axis plays a role in regulation of preadipocyte proliferation, which will contribute to further revealing the mechanisms underlying fat accumulation.

## SUPPLEMENTARY MATERIAL

**Supplementary Table S1**. Information for siRNA sequence against PABPN1 **Supplementary Table S2**. Primers used for real-time quantitative PCR **Supplementary Table S3**. Oligonucleotides used for electrophoretic mobility shift assay

**Supplementary Figure S1**. Efficiencies of overexpression vector and siRNA against PABPN1. **Supplementary Figure S2**. Effi-ciencies of overexpression vector and siRNA against CREB.
