# Supplementary material for: PABPN1 functions as a downstream gene of CREB to inhibit the proliferation of preadipocytes
Source: Anim Biosci. 2024 Aug 26;38(1):41–53. doi: 10.5713/ab.24.0072 (PMC11725739; doi:10.5713/ab.24.0072)
Supplement: Supplementary file 1 [file ab-24-0072-Supplementary-Table.pdf]

Table S1 Sequences of siRNAs against PABPN1 and CREB

Names	Sequence (5’-3’)
siRNA PABPN1-651 (mouse)	AUAUAGAGUUCUCGGACAATT
	UUGUCCGAGAACUCUAUAUTT
Negative control	UUCUCCGAACGUGUCACGUTT
	ACGUGACACGUUCGGAGAATT
siRNA PABPN1-435 (pig)	GUAGAGAAGCAGAUGAAUATT
	UAUUCAUCUGCUUCUCUACTT
siRNA CREB-806 (mouse+pig)	AGACAUUAACCAUGACCAATT
	UUGGUCAUGGUUAAUGUCUTT

Table S2 Primers used for real-time quantitative PCR and CHIP-qPCR

Genes	Sequence (5’-3’)
pig-PABPN1	F: CCCAAAACGAACCAACAGACC
	R: GGGGAATACCATGATGTCGCT
pig-CREB	F: CTCCGGAACTCAGATTTCAACTA
	R: TCCTGGTGCGTCAGAAGATAA
pig-β-actin	F: CTGAAAGCAGAGCCTAATCC
	R: GGCAGGGTCAAGAGTGGTG
mouse-CCND1	F: CGTATCTTACTTCAAGTGCGTG
	R: ATGGTCTCCTTCATCTTAGAGG
mouse-CCNE1	F: CAGAGCAGCGAGCAGGAGC
	R: GCAGCTGCTTCCACACCACT
mouse-β-actin	F: TGTCACGCACGATTTCC
	R: CCCATCTACGAGGGCTAT
mouse-CDK4	F: GAGTGTGAGAGTTCCTAATGGA
	R: GGTCCTGGTCTATATGCTCAAA
mouse-PABPN1	F: CCGCTCTATCTACGTTGGC
	R: GTTGGTTCGTTTGGGAATCAC
mouse-PCNA	F: GAAGTTTTCTGCAAGTGGAGAG
	R: CAGGCTCATTCATCTCTATGGT
mouse-CREB	F: ACCATTGCCCCTGGAGTT
	R: CCTGTTCTTCATTAGACGGACC
CREB	F: TCGGTGATGGTGTTGCTG
	R: TGCGATGTCGTCCTTGTG
PABPN1-5’UTR-CREB	F: GGTCCTTTCCACTCGCTCAG
(CHIP-qPCR)	R: GCCCTTAGGTAACAATGCCG

Table S3 Oligonucleotides used for electrophoretic mobility shift assay

Names	Sequence
Bio-CREB-F	bio-AAGCAAGAACGTCGTCACAGCGTGG-bio
CREB-F	AAGCAAGAACGTCGTCACAGCGTGG
CREB-R	CCACGCTGTGACGACGTTCTTGCTT
CREB-M-F	AAGCAAGAGATCTACTGCAGCGTGG
CREB-M-R	CCACGCTGCAGTAGATCTCTTGCTT
