# Supplementary material for: PABPN1 functions as a downstream gene of CREB to inhibit the proliferation of preadipocytes
Source: Anim Biosci. 2024 Aug 26;38(1):41–53. doi: 10.5713/ab.24.0072 (PMC11725739; doi:10.5713/ab.24.0072)

Figure S1. Efficiencies of overexpression vector and siRNA against PABPN1. A, Efficiencies of vectors overexpressing PABPN1; B, Efficiencies of siRNA against PABPN1 in porcine preadipocytes; C, Efficiencies of siRNA against PABPN1 in 3T3-L1 cells. The results were expressed as mean ± SEM. (n = 3). ** mean $p < 0.01$.

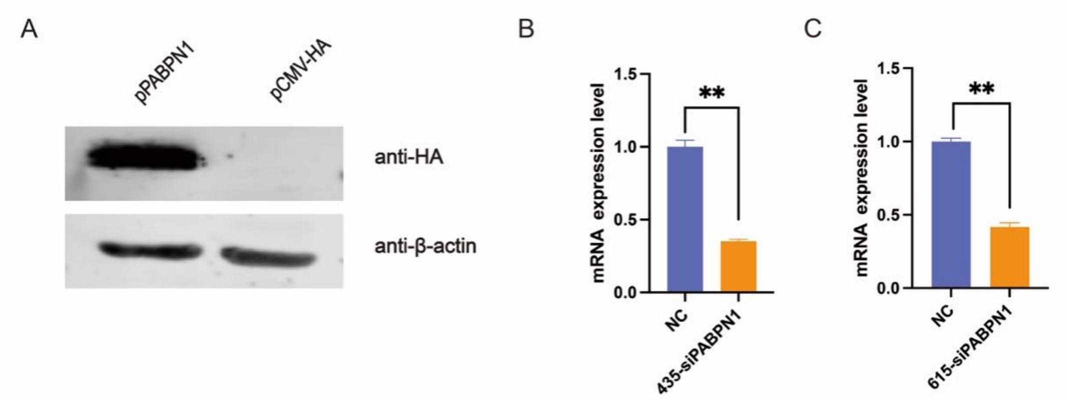

Figure S2. Efficiencies of overexpression vector and siRNA against CREB. A, Efficiencies of vectors overexpressing CREB; B, Efficiencies of siRNA against CREB in porcine preadipocytes; C, Efficiencies of siRNA against CREB in 3T3-L1 cells. The results were expressed as mean ± SEM. (n = 3). ** mean $p < 0.01$.

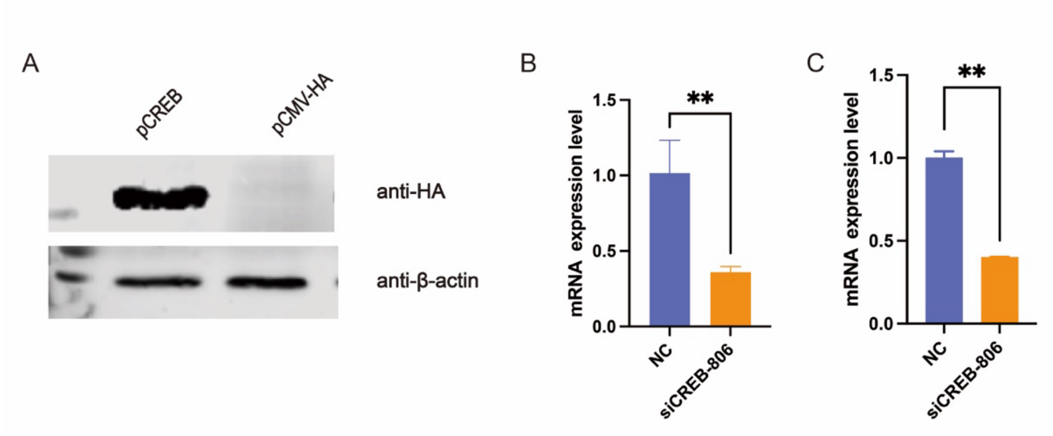

Supplement: Supplementary file 2 [file ab-24-0072-Supplementary-Fig.pdf]
